# Genetic Modifiers of Hereditary Neuromuscular Disorders and Cardiomyopathy

**DOI:** 10.3390/cells10020349

**Published:** 2021-02-08

**Authors:** Sholeh Bazrafshan, Hani Kushlaf, Mashhood Kakroo, John Quinlan, Richard C. Becker, Sakthivel Sadayappan

**Affiliations:** 1 Heart, Lung and Vascular Institute, Division of Cardiovascular Health and Disease, Department of Internal Medicine, University of Cincinnati College of Medicine, Cincinnati, OH 45267, USA; sh.bazrafshan.k@gmail.com (S.B.); kakroomd@ucmail.uc.edu (M.K.); BECKERRC@ucmail.uc.edu (R.C.B.); 2 Department of Neurology and Rehabilitation Medicine, Neuromuscular Center, University of Cincinnati Gardner Neuroscience Institute, University of Cincinnati College of Medicine, Cincinnati, OH 45267, USA; KUSHLAHA@ucmail.uc.edu (H.K.); QUINLAJG@UCMAIL.UC.EDU (J.Q.)

**Keywords:** neuromuscular disorders, muscular dystrophy, cardiomyopathy, genotype–phenotype relationships, next-generation sequencing

## Abstract

Novel genetic variants exist in patients with hereditary neuromuscular disorders (NMD), including muscular dystrophy. These patients also develop cardiac manifestations. However, the association between these gene variants and cardiac abnormalities is understudied. To determine genetic modifiers and features of cardiac disease in NMD patients, we have reviewed electronic medical records of 651 patients referred to the Muscular Dystrophy Association Care Center at the University of Cincinnati and characterized the clinical phenotype of 14 patients correlating with their next-generation sequencing data. The data were retrieved from the electronic medical records of the 14 patients included in the current study and comprised neurologic and cardiac phenotype and genetic reports which included comparative genomic hybridization array and NGS. Novel associations were uncovered in the following eight patients diagnosed with Limb-girdle Muscular Dystrophy, Bethlem Myopathy, Necrotizing Myopathy, Charcot-Marie-Tooth Disease, Peripheral Polyneuropathy, and Valosin-containing Protein-related Myopathy. Mutations in COL6A1, COL6A3, SGCA, SYNE1, FKTN, PLEKHG5, ANO5, and SMCHD1 genes were the most common, and the associated cardiac features included bundle branch blocks, ventricular chamber dilation, septal thickening, and increased outflow track gradients. Our observations suggest that features of cardiac disease and modifying gene mutations in patients with NMD require further investigation to better characterize genotype–phenotype relationships.

## 1. Introduction

One in every 1000 individuals in the world is affected by hereditary neuromuscular disorders (NMD), including muscular dystrophy (MD). The prevalence of NMD varies in different geographic regions and ethnic groups. In a nationwide study in New Zealand, the reported age-standardized prevalence was 22.3 per 100,000 and two-fold higher among individuals of European ancestry compared to Māori, Pasifika, and Asian ethnic groups [1]. In the northern Navarre region of Spain, the prevalence of NMD was as high as 59 per 100,000 individuals [1]. For all types of Charcot-Marie-Tooth disease (CMT), this measure was 15.7 per 100,000, including the highest number of 6.9 per 100,000 reported for CMT type 1A [2]. Over 40 genes have been linked to MD which comprises a group of disorders with muscle weakness and atrophy that can be inherited in an autosomal dominant or recessive, X-linked, or sporadic mode [3,4].

The worldwide pooled prevalence of combined MD per 100,000 is 16.14, including 8.26 for myotonic dystrophy, 3.95 for Facioscapulohumeral dystrophy, 1.63 for Limb-girdle muscular dystrophy (LGMD), and 0.99 for congenital muscular dystrophies, while this number is 4.78 for Duchenne muscular dystrophy and 1.53 for Becker muscular dystrophy (BMD) per 100,000 males [5,6]. Depending on the type of MD and the stage of the disease, cardiac manifestations can vary from none to episodes of sinus tachycardia, arrhythmias, cardiomyopathies, heart failure (HF), and sudden cardiac death (SCD) [7,8,9,10,11]. Cardiomyopathy is one of the most prevalent cardiac phenotypes in patients with MD, of which dilated cardiomyopathy (DCM) is the most common. Cases of left ventricular non-compaction cardiomyopathy and hypertrophic cardiomyopathy (HCM) are also found [12,13,14]. Owing to the advances in next-generation sequencing (NGS), novel gene mutations have been found in NMD, including different types of MD patients with severe cardiac and cardiomyopathy phenotypic features [15,16].

Using Whole exome sequencing, Chardon and colleagues reported shared compound mutations in siblings with childhood-onset severe LGMD, as well as biventricular dilatation and systolic dysfunction [15]. The siblings had inherited heterozygous missense mutations in exon 5 (c.C356T and c.C342G) and exon 11 (c.T1034C) of LIM zinc finger domain containing 2 (*LIMS2*) gene [15]. In a four-generation family with BMD, 73 members had severe cardiomyopathy and HF. A filtering gene strategy revealed a co-segregated novel in-frame mutation (c.4998_5000 del CAG, p.1667 del Ala) in the dystrophin (*DMD*) gene which was associated with cardiomyopathies and fibro fatty replacement of cardiomyocytes in the left ventricle (LV) [17]. Another family was described with two male BMD siblings whose skeletal muscle function was preserved and whose cardiac phenotype demonstrated early onset severe DCM and progressive HF [18]. The genetic study in one sibling uncovered a novel frameshift mutation in exon 27 of *DMD* gene (c.3779_3785delCTTTGGAins GG), translating to one amino acid substitution and early termination (p.Thr1260Argfs*8) [18]. Further, NGS-based analysis revealed a novel missense (c.C7525T) mosaic mutation in *DMD* gene in a 44-year-old male with asymmetric muscle weakness and DCM with reduced ejection fraction (EF) [16]. Sanger sequencing of X chromosome exomes was performed in multiple generations of a Chinese family with Emery-Dreifuss muscular dystrophy. This analysis also uncovered cardiac conduction abnormalities and a high incidence of SCD [19]. The results identified a novel duplication mutation (c.405dup/p.Asp136X) in emerin gene in this family, suggesting the necessity of testing for emerin gene mutations in patients with cardiac conduction disease and SCD of X-linked inheritance with and without neuromuscular phenotypic features [19].

However, it is unclear whether isolated novel mutations or compound gene mutations underlie cardiomyopathies or define the severity of specific cardiac phenotypes. Therefore, further investigation and genotype–phenotype correlations are required to determine optimal risk stratification, surveillance, and management in NMD patients. In the present retrospective study, we aimed to determine the presence of modifying novel and rare genetic variants and describe genotype–phenotype parameters in patients with NMD. Our study determined several known and several not previously described variants of clinical significance associated with cardiac dysfunction and cardiomyopathies. Our findings suggest that NGS of selected gene panels, or even whole genome sequencing, should be included as a routine genetic diagnostic tool to determine the presence of polygenic mutations in several genes.

## 2. Methods

The Institutional Review Board of the University of Cincinnati approved this retrospective observational study (13 December 2018; Institutional Review Board ID # 2018-8124, Title: Genetic modifiers of Muscular Dystrophy and Cardiomyopathies), and all staff members disclosed their respective conflicts of interest. The data were retrieved from electronic medical records (EMR) of patients referred to Muscular Dystrophy Association (MDA) Care Center at University of Cincinnati Medical Center (UCMC) up to November 2018. The subjects were included if they (1) were at least 18 years of age, (2) had one code of International Classification of Diseases-10th Revision for MD disorders, and (3) had one echocardiography or cardiac magnetic resonance imaging on file. Inclusion and exclusion criteria were established, and a request was formally submitted to the Center for Health Information at the Department of Biomedical Informatics at The University of Cincinnati College of Medicine. The Center for Health Information, which is a broker with access to the information of UCMC patients, retrieved the requested data from the Electronic Privacy Information Center, an EMR system. Authorized researchers listed in the approved protocol for collecting genetic and relevant clinical information performed a detailed chart review. Finally, the collected data were stored in Research Electronic Data Capture, which is a mature, secure web application for building and managing online surveys and databases.

Genetic testing panels were obtained as part of the clinical evaluation of patients. Type of the genetic analysis, number of genes in each panel, and detailed information on each reported variant, were imported in an excel sheet in a deidentified manner. The genetic tests and specific panels were already requested by the treating physician as part of clinical assessment, and the results were uploaded in the patients’ EMR database where we accessed information for the present study. The diagnostic genetic panels were as follows: Custom Limb-Girdle Muscular Dystrophy Panel (Subjects # 1, 2, 3, 4, 6, 8, 10, 11, 12, 13, and 14, EGL Genetics, Tucker, GA, USA), Invitae Comprehensive Neuromuscular Disorders Panel (Subject # 4, Invitae, San Francisco, CA, USA), Invitae Comprehensive Neuropathies Panel (Subjects # 5 and 7, Invitae), Expanded Neuromuscular Disorders: Deletion/Duplication Panel (Subjects # 9, EGL Genetics), and Comprehensive Neuromuscular Disorder Panel (Subjects # 10, PerkinElmer, Hebron, KY, USA). Comparative genomic hybridization array was used for subject # 9; otherwise, NGS was used for the other subjects. The name of the genes tested in each panel have been listed in Supplementary Materials. ClinVar, PubMed, OMIM, and EGL Genetics databases were queried for reported variants and mutations. Minor allele frequencies were obtained from NCBI website [20], based on the reports from multiple databases such as Genome Aggregation Database, Trans-Omics for Precision Medicine, and Exome Aggregation Consortium. As part of exploratory analysis, descriptive statistics and frequency tables were created for demographic and genetic characteristics, as well as cardiac phenotype.

## 3. Results

**Figure 1:**
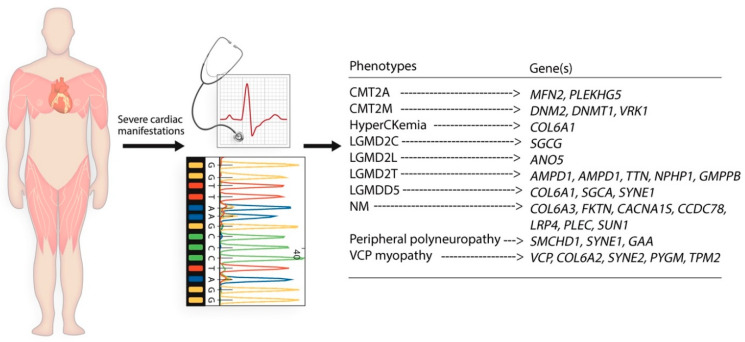
A schematic diagram demonstrates the outline of the retrospective studies defining different types of NMD and different genetic variants. Abbreviations: *AMPD1* = Adenosine Monophosphate Deaminase 1; *ANO5* = Anoctamin 5; *CACNA1S* = Calcium Voltage-gated Channel Subunit Alpha1 S; *CCDC78* = Coiled-coil Domain Containing 78; CMT2A = Charcot-Marie-Tooth Type 2A; CMT2M = Charcot-Marie-Tooth Type 2M; *COL6A1* = Collagen Type VI Alpha 1 Chain; *COL6A2* = Collagen Type VI Alpha 2 Chain; *COL6A3* = Collagen Type VI Alpha 3 Chain; *DNM2* = Dynamin 2; *DNMT1* = DNA Methyltransferase 1; *DYSF* = Dysferlin; *FKTN* = Fukutin; *GMPPB* = GDP-Mannose Pyrophosphorylase B; LGMD2C = Limb-Girdle Muscular Dystrophy Type 2C; LGMD2L = Limb-Girdle Muscular Dystrophy Type 2L; LGMD2T = Limb-Girdle Muscular Dystrophy Type 2T; LGMDD5 = Limb-Girdle Muscular Dystrophy D5/Bethlem Myopathy; *LRP4* = LDL Receptor Related Protein 4; *MFN2* = Mitofusin 2; NM = Necrotizing Myopathy; *NPHP1* = Nephrocystin 1; *PLEC* = Plectin; *PLEKHG5* = Pleckstrin Homology and RhoGEF Domain Containing G5; *PYGM* = Glycogen Phosphorylase, Muscle Associated; *SGCA* = Sarcoglycan Alpha; *SGCB* = Sarcoglycan Beta; *SGCG* = Sarcoglycan Gamma; *SMCHD1* = Structural Maintenance of Chromosomes Flexible Hinge Domain Containing 1; *SUN1* = Sad1 and UNC84 Domain Containing 1; *SYNE1* = Spectrin Repeat Containing Nuclear Envelope Protein 1; *SYNE2* = Spectrin Repeat Containing Nuclear Envelope Protein 2; *TPM2* = Tropomyosin 2; *TTN* = Titin; *VCP* = Valosin Containing Protein; VCP myopathy = Mutant Valosin Containing Protein Related Myopathy; *VRK1* = VRK Serine/Threonine Kinase 1.

To determine novel genetic markers and their association with cardiac conditions in patients with NMD, we reviewed EMR of 651 patients referred to the MDA Care Center at UCMC. We identified 43 patients who had either a physical copy or a physician’s note of their genetic test results. Of 43 patients, 29 had an incomplete genetic report or a test methodology other than NGS. The remaining 14 patients (age = 44 ± 13.77 years, female = 50%) with NGS genetic reports on file were included in this study (Table 1 and Figure 1). Table 2 and Table 3 summarize the clinical and genetic characteristics of these patients, respectively.

### Cases with NMD in Present Study

Case 1 was a 75-year-old female with history of hyperlipidemia and coronary artery disease who presented with myalgia and elevated creatine kinase (CK) of unknown duration. Investigation through muscle biopsy showed evidence of type IIb muscle fiber atrophy, but no evidence of myopathy; however, NGS confirmed the diagnosis of Limb-Girdle muscular dystrophy type 2L (LGMD2L). Baseline transthoracic echocardiography (TTE) at age 74 years showed grade 1 diastolic dysfunction, trivial aortic and pulmonic regurgitation, and mild tricuspid regurgitation. At age 77 years, she presented with palpitations, dyspnea, first-degree atrioventricular (AV) block, left axis deviation, and features of remote ST-segment elevation myocardial infarction in addition to mild concentric left ventricular hypertrophy (LVH) and mild aortic, pulmonic, and tricuspid regurgitation. NGS-based testing in the patient revealed heterozygous carrier status of two pathogenic mutations in anoctamin 5 (*ANO5*) gene (c.191dupA and c.692G>T) that have been linked to late-onset DCM [21].

Case 2 was a 33-year-old female affected by Limb-Girdle muscular dystrophy type 2C (LGMD2C) with mild focal basal septal hypertrophy and asymmetric septal hypertrophy first reported in TTE at age 21. Multiple ECGs uncovered sinus tachycardia and short PR interval, as well as incomplete right bundle branch block (RBBB) at age 27. According to the NGS testing result, the patient was a heterozygous carrier of two pathogenic mutations in sarcoglycan gamma (*SGCG*) gene (c.195+4_l95+7delAGTA and c.452_458delTTACTGT), indicating the relevance of these findings with prior reports of familial and sporadic DCM in patients with mutations in sarcoglycan genes, including *SGCG* [22,23,24,25,26].

Case 3 was a 50-year-old male patient with proximal and distal muscle atrophy, absent or diminished reflexes in both upper and lower extremities, and flexion contractures of fingers, all compatible with Bethlem myopathy also known as Limb-Girdle muscular dystrophy D5 (LGMDD5). His initial presentations were delayed motor development and “toe-walking” at age 3 years, which necessitated the use of crutches and wheelchair at ages 11 and 42 years, respectively. Cardiac manifestations included palpitation, an incomplete RBBB, and early repolarization. Diminished LV internal diameter in systole and diastole was detected in TTE. The patient was a carrier of the following heterozygous missense variants of unknown significance (VUS): (1) collagen type VI alpha 1 chain (*COL6A1*) gene (c.956A>G), (2) sarcoglycan alpha (*SGCA*) gene (c.155T>G), and 3) spectrin repeat containing nuclear envelope protein 1 (*SYNE1*) gene (c.17342G>A), all biomarkers of DCM [27,28,29].

Case 4 was a 61-year-old male with necrotizing myopathy (NM) who experienced intermittent episodes of rhabdomyolysis at age 51 years and hyperCKemia. The ECG findings included sinus bradycardia, first-degree AV block, and right axis deviation at ages 46 and 62 years, respectively, as well as LVH. The patient was a heterozygous carrier of missense VUS in collagen type VI alpha 3 chain (*COL6A3*) gene (c.1214T>C), a reported biomarker of DCM [29], and fukutin (*FKTN*) gene (c.-7C>G), associated with both DCM and myopathy [29,30]. Further testing for deletion/duplication and sequencing for a comprehensive panel of NMD revealed another missense VUS in calcium voltage-gated channel subunit alpha1 S (*CACNA1S*) gene (c.3398T>C), which we believe may explain the findings of NM and hyperCKemia in this patient [31]. Additional missense VUSs were reported in the following genes: coiled-coil domain containing 78 (*CCDC78*) gene (c.416C>T), LDL receptor related protein 4 (*LRP4*) gene (c.5417_5419delAGA), plectin (*PLEC*) gene (c.4666C>T), and Sad1 and UNC84 domain containing 1 (*SUN1*) gene (c.13C>T).

Case 5 was a 30-year-old woman with Charcot-Marie-Tooth Disease type 2A (CMT2A) and sinus tachycardia. She had inherited one heterozygous missense VUS in pleckstrin homology and RhoGEF domain containing G5 (*PLEKHG5*) gene (c.274G>A) and one pathogenic mutation in mitofusin 2 (*MFN2*) gene (c.839G>A). Mutations in the guanosine triphosphatase region of *MFN2* are known to be associated with CMT2A [32]; however, mutations in the heptad repeat domain have been hypothesized to affect other organs, including heart [33], considering reported cardiac hypertrophy and enlargement in *MFN2* gene-deficient mice [34,35].

Case 6 was a 48-year-old male with a history of chronic weakness and severe sensorimotor peripheral polyneuropathy. He demonstrated no evidence of myopathy or neuromuscular junction disorders in either electromyography or muscle biopsy. Cardiac magnetic resonance imaging at age 48 years revealed mildly enlarged left atrium, mild mitral regurgitation, and mild hypokinesis of the basal and mid septum. The patient was a heterozygous carrier of missense VUS in structural maintenance of chromosomes flexible hinge domain containing 1 (*SMCHD1*) gene (c.3596T>C) and *SYNE1* gene (c.17140G>A). Other novel mutations in both genes have already been reported in patients with cardiomyopathies [28,36], supporting the relevance of currently reported mutations with the cardiac phenotype in this patient.

Case 7 was a 57-year-old woman with Charcot-Marie-Tooth Disease type 2M (CMT2M) and a history of ptosis, diplopia and evidence of chronic and severe sensorimotor axonal polyneuropathy, but no evidence of myopathy. Echocardiography and ECG findings at ages 46 and 56 years, respectively, were normal. The patient was a heterozygous carrier of VUS in DNA methyltransferase 1 (*DNMT1*) gene (c.1043C> T), and VRK serine/threonine kinase **1** (*VRK1*) gene (c.8G>A), in addition to dynamin 2 (*DNM2*) gene (c.2576_2578delCCA), which is associated with severe cardiomyopathies [37].

Case 8 was a 30-year-old female with a history of elevated CK, systemic lupus erythematosus, episodes of palpitation, T wave inversion in ECG, and a normal TTE. The NGS-based testing for MD panel showed a heterozygous carrier status for a missense VUS in *COL6A1* gene (c. 1273-4T>C).

Case 9 was a 31-year-old female who initially presented with an elevated CK and muscle biopsy consistent with MD, but normal TTE. Genetic evaluation revealed her to be a heterozygous carrier for missense mutations in adenosine monophosphate deaminase 1 (*AMPD1*), titin (*TTN*), and nephrocystin 1 (*NPHP1*) genes. Further genetic investigation indicated one pathogenic and one VUS in GDP-mannose pyrophosphorylase B (*GMPPB*) gene and confirmed the diagnosis of Limb-Girdle muscular dystrophy type 2T (LGMD2T).

Case 10 was a 49-year-old man who initially presented with progressive muscle weakness in upper and lower extremities in his late 30s with no sensory manifestations or incontinence. His father had a history of myopathy, dementia, and heart attack in his 40s. Deltoid muscle biopsy revealed chronic myopathy with rimmed vacuoles and mild denervation atrophy. Echocardiography at age 45 years showed diastolic flattening of the ventricular septum and high septal fractional thickening, as well as high aortic and pulmonic valve peak velocities. Initial genetic testing identified that he was a heterozygous carrier of missense VUS in collagen type VI alpha 2 chain (*COL6A2*) gene (c.1970-3C>A) and spectrin repeat containing nuclear envelope protein 2 (*SYNE2*) gene (c.17539G>A). Further genetic sequencing revealed missense VUS in the glycogen phosphorylase, muscle associated (*PYGM*) gene (c.580C>T) and tropomyosin 2 (*TPM2*) gene (c.536C>T), in addition to a missense pathogenic mutation in the valosin containing protein (*VCP*) gene, confirming the diagnosis of VCP myopathy. Missense mutations in the *VCP* gene have been associated with clinical variability, including myopathy and cardiomyopathy [38].

Cases 11, 12, 13, and 14 were evaluated for NMD; however, their clinical presentations and investigations were all inconclusive. Case 11 was a 53-year-old male with elevated CK, chronic myopathy, axonal sensorimotor peripheral neuropathy, mild LVH, and diastolic dysfunction grade 1 in echocardiography, in addition to NGS that revealed one heterozygous missense VUS in dysferlin (*DYSF*) gene (c.3213C>T). Case 12 was a 45-year-old DCM male whose muscle biopsy was indicative of dystrophic changes with no evidence of inflammation, in addition to elevated CK, diffuse myopathy in electromyography, and heterozygous carrier status of a missense VUS in structural *SMCHD1* gene (c.4787G>A). This patient’s clinical presentations were found to be compatible with BMD but had not been confirmed with genetic analysis at the time of this report. Case 13 was a 25-year-old female evaluated for elevated CK, multiple episodes of rhabdomyolysis, inconclusive electromyography and muscle biopsy, and normal cardiac findings. Further genetic study showed that she was a heterozygous carrier for missense VUS in *PLEC*, sarcoglycan beta (*SGCB*), and *TTN* genes. Case 14 was a 45-year-old male with elevated CK, one episode of rhabdomyolysis, and mild LVH in echocardiography. NGS-based testing for LGMD panel showed no mutation.

## 4. Discussion

Patients with hereditary NMD can carry novel genetic variants, leading to different cardiac outcomes. Our retrospective correlation study of a cohort of NMD patients suggests that selected gene panels derived from NGS, or even whole genome sequencing, should be included as part of a routine genetic screening to determine the presence of polygenic mutations associated with cardiovascular disease. Diagnosing and presenting a full range of clinical phenotypes should support a patient-specific management approach that involves a multidisciplinary team, for instance, a genetic counselor, cardiologist, pulmonologist, and a physiotherapist.

Late onset myopathy and asymptomatic isolated elevated CK have been reported in patients with homozygous mutation in *ANO5* encoding the anoctamin protein family which mainly acts as a calcium-activated chloride channel and is expressed in both skeletal and cardiac muscles [39]. Mutations in *ANO5* with recessive inheritance occur in proximal LGMD2L and Miyoshi muscular dystrophy 3 (MMD3) [40,41]. In 2013, Wahbi and colleagues performed a comprehensive analysis of cardiac involvement in 19 patients from 16 families with isolated elevated CK or myopathy and a proven mutation in *ANO5* [21]. They reported a 30-year-old MMD3 male patient with missense mutation (c.1898+1G>A) and ECG findings of ventricular premature beats without tachycardia, LVH, early repolarization, and left anterior fascicular block, in addition to a confirmed DCM. They also reported a 34-year-old male with isolated elevated CK and a missense mutation (c.1733T>C) [21]. Here, we identified a 75-year-old LGMD2L female with elevated CK, history of coronary artery disease, and carrier status of 2 heterozygous mutations in *ANO5*. She also presented with palpitations and dyspnea. A first-degree AV block, left axis deviation, remote ST-segment elevation myocardial infarction, mild concentric LVH and mild aortic, pulmonic, and tricuspid regurgitation were uncovered. In Wahbi’s study, these same mutations were reported in a 47-year-old female with isolated elevated CK, but no cardiac manifestations [21].

Mutations in α-, β-, γ-, and δ-sarcoglycans with sporadic and familial cardiomyopathy do occur [24,26]. Sarcoglycans belong to the dystrophin-associated glycoprotein complex [42,43] and are associated with early onset MD [44,45,46,47,48]. The *SGCG* gene, which is linked to chromosome 13 and autosomal recessive inheritance [46,49], is associated with interventricular septum abnormalities, as reported in a young Dutch female homozygous carrier of a novel missense mutation in the D13S232 region near *SGCG* gene. The mutation affected the members of a Dutch family with childhood onset and slowly progressive MD [50]. Cardiomyocytes isolated from LGMD2C mice lacking γ-sarcoglycan have normal function versus cardiomyocytes isolated from dystrophin-deficient mice [51]. Our case series included a c.195+4_l95+7delAGTA deletion in intron 2 and c.452_458delTTACTGT deletion in exon 5 of *SGCG* in a LGMD2C female with mild focal basal septal hypertrophy, asymmetric septal hypertrophy and sinus tachycardia, short PR interval, and incomplete RBBB. A four-base-pair deletion in the splice site of intron 2 was reported in a 10-year-old homozygous female carrier and 2 heterozygous female carriers aged 14 and 24 years who were all diagnosed with LGMD2C, but who had normal ECG and TTE findings [52]. Further in vivo and in vitro research is needed to determine the effect of this heterozygous compound mutation on cardiomyocytes.

Our cases included a patient with LGMDD5 accompanied by palpitations, incomplete RBBB, early repolarization and decreased LV internal diameter during systole and diastole. He was a heterozygous carrier of 3 VUS: c.956A>G in *COL6A1*, c.155T>G in *SGCA*, and c.17342G>A in *SYNE1*. Variable phenotypic features in LGMDD5 have been associated with mutations in different locations of genes encoding collagen VI [53], including *COL6A1*, one of the introduced biomarkers of DCM in a human induced pluripotent stem cell-derived cardiomyocyte model [29]. De novo heterozygous missense mutation c.956A>G in *COL6A1* was first reported in a Japanese male patient with sarcolemma-specific collagen VI deficiency, but no cardiac findings [54]. However, in a cardiac evaluation of 50 LGMDD5 patients of 26 families, van der Kooi and colleagues reported one 37-year-old Dutch male with simultaneous mutations in *COL6A2* and *SNC5A*. He presented with Brugada syndrome–like electrocardiographic findings with incomplete RBBB and ST-segment elevation in the right precordial leads. The authors concluded that none of the reported cardiac findings was related to the corresponding LGMDD5 [55]. As described earlier, sarcoglycan genes are associated with dystrophin and cardiomyopathies [22,24,26,56], including *SGCA* which encodes α component and was reported in relation to mild cardiomyopathies in the α-sarcoglycan-deficient mouse model [55]. In addition, novel mutations in *SYNE1* have also been linked to both HCM [28] and DCM [57]. Thus, further cellular and animal model investigations are required to characterize the effects of reported missense mutations in *COL6A1*, *SGCA*, and *SYNE1* in the current patient’s cardiovascular system.

In our population, two heterozygous missense VUSs in *COL6A3* gene (c.1214T>C) and *FKTN* gene (c.-7C>G) were reported in a 61-year-old male with NM with sinus bradycardia, first degree AV block, and right axis deviation in ECG, as well as LVH in TTE. Controversy exists regarding the pathogenicity of *COL6A3* (c.1214T>C) missense variant, which has already been reported in a case of LGMDD5 [58]. However, the presence of this variant in the current patient with skeletal and cardiac muscle involvement mandates further investigation. Notably, *COL6A3* gene is a potential biomarker of DCM in the human induced pluripotent stem cell-derived cardiomyocyte model, as described by Zhuang and colleagues [29], as well as DCM cases reported in carriers of *FKTN* mutation, the gene linked to Fukuyama-type congenital MD [30].

Clinical and histologic manifestations of VCP myopathy were first described in 1982 [59] and then in 2000 [60] and 2001 [61]. Researchers observed five different families whose members suffered from a unique autosomal dominant disorder with variable penetrance of inclusion body myopathy related to Paget’s disease of bone, fronto-temporal dementia, and cardiomyopathies. Despite late-onset presentation, most patients die in their 40s to 60s owing to progressive muscle weakness and both respiratory and cardiac failure [60]. The role of VCP in the pathogenesis of cardiomyopathy has been studied with a mutant VCP-overexpressing mouse model lacking adenosine triphosphatase activity, which resulted in the development of cardiomyopathy [62]. The cooperative role of *COL6A2* and DS cell adhesion molecule (*DSCAM*) genes in the pathogenesis of congenital heart defects has been studied with both in vivo and in vitro models [63]. The nesprin-2 encoding gene *SYNE2* has also been reported in patients with cardiomyopathy phenotype and inheritance status of multiple variants of unknown significance [57,64]. *PYGM* gene which encodes myophosphorylase enzyme was also related to HCM [65], as well as *TPM2* gene, which was reported in patients with myopathy and DCM. The current patient is a heterozygous carrier of multiple genetic variants of unknown significance, but also presents with diastolic flattening of ventricular septum contour and high septal fractional shortening. These genes include *VCP*, *COL6A2*, *SYNE2*, *PYGM* gene, and *TPM2*, and they warrant investigation of the more complex interacting mechanism underlying the hereditary cardiomyopathy phenotypic features.

Indeed, advancement in genetic testing techniques facilitated discovery of novel variants in different genes in NMD patients with cardiomyopathy phenotypes. However, our knowledge on the pathogenic role of these variants is still little. For example, mutations in desmin encoding gene (*DES*) were reported in association with myopathies in both skeletal and cardiac muscles leading to the development of in vitro and in vivo tools to widely investigate these mutations including the novel variants and their clinical impact [66]. Understanding and classifying the newly found variants are essential in early detection of patients through genetic screening.

### Study Limitation

The present retrospective and descriptive pilot study comprised a small sample size of 14 subjects with different types of NMD and different genetic variants. Having subjects with the same phenotype tested with the same genetic panel would contribute greater insights for the development of NMD. This limitation precluded us from performing a systematic genotype–phenotype analysis and correlation, thus necessitating future retrospective or prospective analytical research with an adequate number of subjects of specific types of NMD and familial history.

## 5. Conclusions

Novel genetic variants exist in patients with hereditary neuromuscular disorders (NMD), including muscular dystrophy. These patients also develop cardiac conditions. The association between these gene variants and cardiac conditions in NMD is understudied. We identified eight patients referred to the Muscular Dystrophy Association Care Center at the University of Cincinnati Medical Center. They were known carriers of the following gene variants: *COL6A1*, *COL6A3*, *SGCA*, *SYNE1*, *FKTN*, *PLEKHG5*, *ANO5* and *SMCHD1*. These same variants have been associated with cardiac conditions, including cardiomyopathy. Based on our findings, we recommend further study of larger cohorts of NMD patients to better determine the features of cardiac disease, modifying gene mutations, and genotype–phenotype relationships.

## Figures and Tables

**Table 1:** Demographic and anamnestic characteristics of patients.

Variables	*n* = 14
Age (year)	44.00 ± 13.77 *
Female, *n* (%)	7 (50)
African-American ethnicity, *n* (%)	3 (21.42)
BMI kg/m^2^	31.02 ± 7.78
BSA m^2^	2.1 ± 0.39
Current smoker, *n* (%)	3 (21.42)
Hypertension, *n* (%)	6 (42.85)
Hyperlipidemia, *n* (%)	3 (21.42)
Diabetes mellitus, *n* (%)	3 (21.42)
Coronary artery disease, *n* (%)	2 (14.28)

Legends: BMI = Body Mass Index; BSA = Body Surface Area. * Age, BMI and BSA have been reported with mean ± SD.

**Table 2:** Echocardiographic characteristics of patients.

	1	2	3	4	5	7	8	9	10	11	12	13	14
Diagnosis	LGMD2L	LGMD2C	LGMDD5	NM	CMT2A	CMT2M	HyperCKemia	LGMD2T	VCP myopathy	Unknown	Unknown	Unknown	Unknown
Baseline echocardiographic characteristics													
LAD/BSA	1.6	1.6	1.53	2.28 ^a^	1.6	1.5	1.7	1.8	1.5	NA	1.6	1.64	1.3
LVES ID mm	26	24	22.3 ^a^	27.15	29	32	23	24.4	28.96	34.73	34	29.49	29
LVED ID mm	39	35	36.7 ^a^	39.94	45	48	41	37.9	49.04	53.17	50	42.68	44
EF%	70	60	77.5	60.72	74	62.5	74	62.5	71.59	63.38	59	58.88	72
FS%	33	31	39	32	36	34	43	36	41	35	31	31	34
Longitudinal strain %	−20.96	−12.9	NA	NA	−18.6	NA	−17.6	NA	NA	NA	−21.05	NA	NA
IVS ED mm	13 ^a^	9.0	10.2	15.99 ^a^	9.0	7.0	0.7	8.1	11.32 ^a^	12.66 ^a^	14 ^a^	11.42 ^a^	12 ^a^
LVPW ED mm	12 ^a^	9.0	10.9	15.48 ^a^	7.0	9.0	8.0	9.2	10.68	11.44 ^a^	10	8.97	14 ^a^
IVS/LVPW	1.08	1.06	0.94	1.03	1.26	0.77	0.96	0.88	1.06	1.11	1.4	1.27	0.9
DD grade	1.0	-	1.0	-	-	-	-	-	-	1.0	1.0	-	-
LVH/HCM/DCM	mild LVH	-	-	mod LVH	-	-	-	-	-	mild LVH	DCM	-	mild LVH
Doppler echocardiographic characteristics													
E/A	1.0	1.26	0.7	1.11	1.44	1.2	2.23	1.89	1.27	0.65	0.72	2.43	1.0
E/e’	14	8.5	6.45	8.75	6.0	6.7	4.72	6.4	6.1	4.6	8.17	6.5	6.0
DT	313	93	243	214	206	217	150	260	240	220	150	264 ^a^	109
AV peak velocity cm/s	130	90	NA	117.86	110	118	100	125.16	98.72	118.22	120	130.94	80
AV mean velocity cm/s	84 ^a^	NA	NA	64.7 ^a^	NA	NA	NA	NA	73.04 ^a^	87.64	NA	NA	NA
PV peak velocity cm/s	100	100	NA	NA	100	52	100	NA	88.79 ^a^	98.31 ^a^	100	52.01 ^a^	200
AR	mild	-	-	-	-	-	-	trivial	-	-	-	-	-
MR	mild	-	-	trivial	-	-	-	trivial	-	-	-	trivial	-
TR	mild	-	-	trivial	-	-	-	trivial	trivial	-	trivial	-	-
PR	mild	-	-	-	-	-	-	trivial	-	-	-	-	trivial

Abbreviations: LAD = Left Atrial Diameter; BSA = Body Surface Area; LVES ID = Left Ventricular Internal Diameter in Systole; LVED ID = Left Ventricular Internal Diameter in Diastole; LV = Left Ventricle; EF = Ejection Fraction; FS = Fractional Shortening; IVS ED = End Diastolic Interventricular Septum; LVPW ED = End Diastolic Left Ventricular Posterior Wall Thickness; DD = Diastolic Dysfunction; LVH = Left Ventricular Hypertrophy; HCM = Hypertrophic Cardiomyopathy; DCM = Dilated Cardiomyopathy; E = Early Mitral Inflow Velocity; A = Peak Mitral Inflow Velocity; e’ = Mitral Annular Early Diastolic Velocity; DT = Deceleration Time; AV = Aortic valve; PV = Pulmonic Valve; AR = Aortic Regurgitation; MR = Mitral Regurgitation; TR = Tricuspid Regurgitation; PR = Pulmonic Regurgitation; LGMD2L = Limb-Girdle Muscular Dystrophy Type 2L; LGMD2C = Limb-Girdle Muscular Dystrophy Type 2C; LGMDD5 = Limb-Girdle Muscular Dystrophy D5/Bethlem Myopathy; NM = Necrotizing Myopathy; CMT2A = Charcot-Marie-Tooth Hereditary Neuropathy Type 2A; CMT2M = Charcot-Marie-Tooth Hereditary Neuropathy Type 2M; HyperCKemia = Persistent Elevation of Serum CK; CK = Creatine Kinase; LGMD2T = Limb-Girdle Muscular Dystrophy Type 2T; VCP myopathy = Mutant Valosin Containing Protein Related Myopathy. NA = Not Available. ^a^ represents values outside reference range specified by echocardiography lab.

**Table 3:** Genetic characteristics of patients.

	Diagnosis	Lab	Variant 1	Variant 2	Variant 3	Variant 4	Variant 5	Variant 6	Variant 7
1	LGMD2L	EGL Genetics	*ANO5*	*ANO5*					
			Exon 5	Exon 8					
			c.191dupA	c.692G>T					
			MAF: dupA = 0.001096	T = 0.00102					
			p.N64KfsX15	p.Gly231Val					
			Duplication, Het	Missense, Het					
			Pathogenic	Pathogenic					
			MIM # 608662						
2	LGMD2C	EGL Genetics	*SGCG*	*SGCG*					
			IVS 2	Exon 5					
			c.195+4_195+7delAGTA	c.452_458delTTACTGT					
			MAF: delAGTA = 0.000008	NA					
			-	p.Leu150_Phe151insTer					
			Deletion, Het	Deletion, Het					
			Pathogenic	Pathogenic					
			MIM # 608896						
3	LGMDD5	EGL Genetics	*COL6A1*	*SGCA*	*SYNE1*				
			Exon 12	Exon 2	Exon 92				
			c.956A>G	c.155T>G	c.17342G>A				
			MAF: NA	G = 0.000036	NA				
			p.Lys319Arg	p.Val52Gly	p.Arg5781His				
			Missense, Het	Missense, Het	Missense, Het				
			VUS	VUS	VUS				
			MIM # 120220	MIM # 600119	MIM # 608441				
4	NM	EGL Genetics & Invitae	*COL6A3*	*FKTN*	*CACNA1S*	*CCDC78*	*LRP4*	*PLEC*	*SUN1*
			Exon 4	5′UTR	Exon 26	Exon 4	Exon 38	Exon 32	Exon 1
			c.1214T>C	c.-7C>G	c.3398T>C	c.416C>T	c.5417_5419delAGA	c.4666C>T	c.13C>T
			MAF: G = 0.00080	G = 0.000004	NA	NA	delTCT = 0.000004	NA	T = 0.000032
			p.Phe405Ser	-	p.Ile1133Thr	p.Ser139Phe	p.Lys1806del	p.Arg1556Cys	p.Arg5Trp
			Missense, Het	Missense, Het	Missense, Het	Missense, Het	Deletion	Missense, Het	Missense, Het
			VUS	VUS	VUS	VUS	VUS	VUS	VUS
			MIM # 120250	MIM # 607440	MIM # 114208	MIM # 614666	MIM # 604270	MIM # 601282	MIM # 607723
5	CMT2A	Invitae	*MFN2*	*PLEKHG5*					
			Exon 9	Exon 5					
			c.839G>A	c.274G>A					
			A = 0.000004	T = 0.000049					
			p.Arg280His	p.Val92Ile					
			Missense, Het	Missense, Het					
			Pathogenic	VUS					
			MIM # 608507	MIM # 611101					
6	Peripheral polyneuropathy	EGL Genetics	*SMCHD1*	*SYNE1*	*GAA*				
			Exon 28	Exon 91	Exon 10				
			c.3596T>C	c.17140G>A	c.1482A>G				
			MAF: NA	NA	G = 0.000080				
			p.Val1199Ala	p.Ala5714Thr	p.Thr494=				
			Missense, Het	Missense, Het	Silent				
			VUS	VUS	VUS				
			MIM # 614982	MIM # 608441	MIM # 606800				
7	CMT2M	Invitae	*DNM2*	*DNMT1*	*VRK1*				
			Exon 21	Exon 14	Exon 2				
			c.2576_2578delCCA	c.1043C>T	c.8G>A				
			MAF: NA	A = 0.000032	NA				
			p.Thr859del	p.Pro348Leu	p.Arg3His				
			Deletion, Het	Missense, Het	Missense, Het				
			VUS	VUS	VUS				
			MIM # 602378	MIM # 126375	MIM # 602168				
8	HyperCKemia	EGL Genetics	*COL6A1*						
			IVS 18						
			c.1273-4T>C						
			MAF: C = 0.000082						
			-						
			Missense, Het						
			VUS						
			MIM # 120220						
9	LGMD2T	EGL Genetics	*AMPD1*	*AMPD1*	*TTN*	*NPHP1*	*GMPPB*		
			Exon 7	Exon 11	Exon 206	Exon 1-20	Report not available		
			c.959A>T	c.1531.A>G	c.40508C>T	Deletion			
			MAF: A = 0.028281	C = 0.000016	NA	NA			
			p.Lys320Ile	p.Met511Val	p.Ser13503Leu	-			
			Missense, Het	Missense, Het	Missense, Het	Deletion, Het			
			Pathogenic	VUS	VUS	Pathogenic			
			MIM # 102770		MIM # 188840	MIM # 607100			
10	VCP myopathy	EGL Genetics	*VCP*	*COL6A2*	*SYNE2*	*PYGM*	*TPM2*		
			Exon 5	IVS 25	Exon 96	Exon 5	Exon 5		
			c.572G>A	c.1970-3C>A	c.17539G>A	c.580C>T	c.536C>T		
			MAF: T = 0.000016	A = 0.00060	A = 0.000096	NA	A = 0.000052		
			p.Arg191Gln	-	p.Glu5847Lys	p.Arg194Trp	p.Ser179Leu		
			Missense, Het	Missense, Het	Missense, Het	Missense, Het	Missense, Het		
			Pathogenic	VUS	VUS	VUS	VUS		
			MIM # 601023	MIM # 120240	MIM # 608442	MIM # 608455	MIM # 190990		
11	Unknown	EGL Genetics	*DYSF*						
			Exon 30						
			c.3213C>T						
			MAF: T = 0.000347						
			p.Tyr1071=						
			Missense, Het						
			VUS						
			MIM # 603,009						
12	Unknown	EGL Genetics	*SMCHD1*						
			Exon 38						
			c.4787G>A						
			MAF: A = 0.000017						
			p.Arg1596Gln						
			Missense, Het						
			VUS						
			MIM # 614982						
13	Unknown	EGL Genetics	*PLEC*	*PLEC*	*SGCB*	*TTN*			
			Exon 32	Exon 32	Exon 1	Exon 224			
			c.4642G>A	c.5412G>C	c.21_23dupGGC	c.44832C>G			
			MAF: T = 0.000086	NA	dupCGC = 0.000462	C = 0.000332			
			p.Val1548Met	p.Glu1804Asp	p.Ala9_Glu10insAla	p.Asn14944Lys			
			Missense, Het	Missense, Het	Duplication, Het	Missense, Het			
			VUS	VUS	VUS	VUS			
			MIM # 601282		MIM # 600900	MIM # 188840			

Abbreviations: *AMPD1* = Adenosine Monophosphate Deaminase 1; *ANO5* = Anoctamin 5; *CACNA1S* = Calcium Voltage-gated Channel Subunit Alpha1 S; *CCDC78* = Coiled-coil Domain Containing 78; CMT2A = Charcot-Marie-Tooth Type 2A; CMT2M = Charcot-Marie-Tooth Type 2M; *COL6A1* = Collagen Type VI Alpha 1 Chain; *COL6A2* = Collagen Type VI Alpha 2 Chain; *COL6A3* = Collagen Type VI Alpha 3 Chain; *DNM2* = Dynamin 2; *DNMT1* = DNA Methyltransferase 1; *DYSF* = Dysferlin; *FKTN* = Fukutin; *GAA* = Alpha Glucosidase; *GMPPB* = GDP-Mannose Pyrophosphorylase B; IVS = Intervening Sequence; LGMD2C = Limb-Girdle Muscular Dystrophy Type 2C; LGMD2L = Limb-Girdle Muscular Dystrophy Type 2L; LGMD2T = Limb-Girdle Muscular Dystrophy Type 2T; LGMDD5 = Limb-Girdle Muscular Dystrophy D5/Bethlem Myopathy; *LRP4* = LDL Receptor Related Protein 4; MAF = Minor Allele Frequency; *MFN2* = Mitofusin 2; NA = Not Available; NM = Necrotizing Myopathy; *NPHP1* = Nephrocystin 1; *PLEC* = Plectin; *PLEKHG5* = Pleckstrin Homology and RhoGEF Domain Containing G5; *PYGM* = Glycogen Phosphorylase, Muscle Associated; *SGCA* = Sarcoglycan Alpha; *SGCB* = Sarcoglycan Beta; *SGCG* = Sarcoglycan Gamma; *SMCHD1* = Structural Maintenance of Chromosomes Flexible Hinge Domain Containing 1; *SUN1* = Sad1 and UNC84 Domain Containing 1; *SYNE1* = Spectrin Repeat Containing Nuclear Envelope Protein 1; *SYNE2* = Spectrin Repeat Containing Nuclear Envelope Protein 2; *TPM2* = Tropomyosin 2; *TTN* = Titin; UTR = Untranslated Region; *VCP* = Valosin Containing Protein; VCP myopathy = Mutant Valosin Containing Protein Related Myopathy; *VRK1* = VRK Serine/Threonine Kinase 1; VUS = Variable of Unknown Significance. OMIM = “Online Mendelian Inheritance in Man (OMIM^®^) is a continuously updated catalog of human genes and genetic disorders and traits, with particular focus on the molecular relationship between genetic variation and phenotypic expression” retrieved from Online Mendelian Inheritance in Man, OMIM^®^. McKusick-Nathans Institute of Genetic Medicine, Johns Hopkins University (Baltimore, MD, USA, 26 June 2020). World Wide Web URL: https://omim.org/ (accessed on 9 November 2020). NCBI website was used for MAF report: https://www.ncbi.nlm.nih.gov/snp (accessed on 9 November 2020).

## Data Availability

The data presented in this study are available on request from the corresponding author. The data are not publicly available due to restrictions e.g., privacy or ethical.

## Supplementary Materials

The following are available online at https://www.mdpi.com/2073-4409/10/2/349/s1. Genetic tests and specific panels that had been obtained as part of the clinical evaluation by a neuromuscular specialist for each clinical case presented in this manuscript.

## Glossary

AMPD1	Adenosine monophosphate deaminase 1
ANO5	Anoctamin 5
AV	Atrioventricular
BMD	Becker muscular dystrophy
CACNA1S	Calcium voltage-gated channel subunit alpha1 S
CCDC78	Coiled-coil domain containing 78
CK	Creatine kinase
CMT	Charcot-Marie-Tooth
CMT2A	Charcot-Marie-Tooth type 2A
CMT2M	Charcot-Marie-Tooth type 2M
COL6A1	Collagen Type VI Alpha 1 chain
COL6A2	Collagen Type VI Alpha 2 chain
COL6A3	Collagen Type VI Alpha 3 chain
DES	Desmin
DCM	Dilated cardiomyopathy
DMD	Dystrophin
DNM2	Dynamin 2
DNMT1	DNA methyltransferase 1
DSCAM	DS cell adhesion molecule
DYSF	Dysferlin
ECG	Electrocardiogram
EF	Ejection fraction
EMR	Electronic medical records
FKTN	Fukutin
GMPPB	GDP-mannose pyrophosphorylase B
HCM	Hypertrophic cardiomyopathy
HF	Heart failure
LGMD	Limb-girdle muscular dystrophy
LGMD2C	Limb-girdle muscular dystrophy type 2C
LGMD2L	Limb-girdle muscular dystrophy type 2L
LGMD2T	Limb-girdle muscular dystrophy type 2T
LGMDD5	Limb-girdle muscular dystrophy D5/Bethlem Myopathy
LRP4	LDL receptor related protein 4
LV	Left ventricle
LVH	Left ventricular hypertrophy
MD	Muscular dystrophy
MDA	Muscular Dystrophy Association
MFN2	Mitofusin 2
MMD	Miyoshi muscular dystrophy
NGS	Next-generation sequencing
NMD	Hereditary neuromuscular disorders
NM	Necrotizing Myopathy
NPHP1	Nephrocystin 1
PLEC	Plectin
PLEKHG5	Pleckstrin homology and RhoGEF domain containing G5
PYGM	Glycogen phosphorylase, muscle associated
RBBB	Right bundle branch block
SCD	Sudden cardiac death
SGCA	Sarcoglycan alpha
SGCB	Sarcoglycan beta
SGCG	Sarcoglycan gamma
SMCHD1	Structural maintenance of chromosomes flexible hinge domain containing 1
SUN1	Sad1 and UNC84 domain containing 1
SYNE1	Spectrin repeat containing nuclear envelope protein 1
SYNE2	Spectrin repeat containing nuclear envelope protein 2
TPM2	Tropomyosin 2
TTE	Transthoracic echocardiography
TTN	Titin
UCMC	University of Cincinnati Medical Center
VCP	Valosin containing protein
VCP myopathy	Mutant valosin containing protein related myopathy
VUS	Variant of unknown significance
VRK1	VRK serine/threonine kinase 1

## References

[1] Pagola-Lorz I., Vicente E., Ibanez B., Torne L., Elizalde-Beiras I., Garcia-Solaesa V., Garcia F., Delfrade J., Jerico I. (2019). Epidemiological study and genetic characterization of inherited muscle diseases in a northern Spanish region. Orphanet J. Rare Dis..

[2] Theadom A., Rodrigues M., Poke G., O’Grady G., Love D., Hammond-Tooke G., Parmar P., Baker R., Feigin V., Jones K. (2019). A Nationwide, Population-Based Prevalence Study of Genetic Muscle Disorders. Neuroepidemiology.

[3] Emery A.E.H. (2002). The muscular dystrophies. Lancet.

[4] Kaplan J.C., Hamroun D. (2015). The 2016 version of the gene table of monogenic neuromuscular disorders (nuclear genome). Neuromuscul. Disord..

[5] Mah J.K., Korngut L., Dykeman J., Day L., Pringsheim T., Jette N. (2014). A systematic review and meta-analysis on the epidemiology of Duchenne and Becker muscular dystrophy. Neuromuscul. Disord..

[6] Mah J.K., Korngut L., Fiest K.M., Dykeman J., Day L.J., Pringsheim T., Jette N. (2016). A Systematic Review and Meta-analysis on the Epidemiology of the Muscular Dystrophies. Can. J. Neurol. Sci..

[7] Blagova O., Nedostup A., Shumakov D., Poptsov V., Shestak A., Zaklyasminskaya E. (2016). Dilated cardiomyopathy with severe arrhythmias in Emery-Dreifuss muscular dystrophy: From ablation to heart transplantation. J. Atr. Fibrillation.

[8] Choudhary P., Nandakumar R., Greig H., Broadhurst P., Dean J., Puranik R., Celermajer D.S., Hillis G.S. (2016). Structural and electrical cardiac abnormalities are prevalent in asymptomatic adults with myotonic dystrophy. Heart.

[9] Kulach A., Majewski M., Gasior Z., Gardas R., Goscinska-Bis K., Golba K.S. (2020). Dilated cardiomyopathy with severe arrhythmias in Emery-Dreifuss muscular dystrophy. Cardiol. J..

[10] Ho R., Nguyen M.L., Mather P. (2016). Cardiomyopathy in becker muscular dystrophy: Overview. World J. Cardiol..

[11] McNally E.M., Kaltman J.R., Benson D.W., Canter C.E., Cripe L.H., Duan D., Finder J.D., Groh W.J., Hoffman E.P., Judge D.P. (2015). Contemporary cardiac issues in Duchenne muscular dystrophy. Working Group of the National Heart, Lung, and Blood Institute in collaboration with Parent Project Muscular Dystrophy. Circulation.

[12] Sveen M.L., Thune J.J., Køber L., Vissing J. (2008). Cardiac involvement in patients with limb-girdle muscular dystrophy type 2 and becker muscular dystrophy. Arch. Neurol..

[13] Tandon A., Taylor M.D., Cripe L.H. (2015). Co-occurring Duchenne muscular dystrophy and hypertrophic cardiomyopathy in an adult with atypical cardiac phenotype. Cardiol. Young.

[14] Feingold B., Mahle W.T., Auerbach S., Clemens P., Domenighetti A.A., Jefferies J.L., Judge D.P., Lal A.K., Markham L.W., Parks W.J. (2017). Management of Cardiac Involvement Associated With Neuromuscular Diseases: A Scientific Statement from the American Heart Association. Circulation.

[15] Chardon J.W., Smith A.C., Woulfe J., Pena E., Rakhra K., Dennie C., Beaulieu C., Huang L., Schwartzentruber J., Hawkins C. (2015). LIMS2 mutations are associated with a novel muscular dystrophy, severe cardiomyopathy and triangular tongues. Clin. Genet..

[16] Ribeiro J., Rebelo O., Fernández-Marmiesse A., Negrão L. (2018). Novel mosaic mutation in the dystrophin gene causing distal asymmetric muscle weakness of the upper limbs and dilated cardiomyopathy. Acta Myol..

[17] Chen L., Ren J., Chen X., Chen K., Rao M., Zhang N., Yu W., Song J. (2018). A novel mutation of dystrophin in a Becker muscular dystrophy family with severe cardiac involvement: From genetics to clinicopathology. Cardiovasc. Pathol..

[18] Tsuda T., Fitzgerald K., Scavena M., Gidding S., Cox M.O., Marks H., Flanigan K.M., Moore S.A. (2015). Early-progressive dilated cardiomyopathy in a family with Becker muscular dystrophy related to a novel frameshift mutation in the dystrophin gene exon 27. J. Hum. Genet..

[19] Kong D., Zhan Y., Liu C., Hu Y., Zhou Y., Luo J., Gu L., Zhou X., Zhang Z. (2019). A Novel Mutation Of The EMD Gene In A Family With Cardiac Conduction Abnormalities And A High Incidence Of Sudden Cardiac Death. Pharmgenom. Pers. Med..

[20] SNP–NCBI. https://www.ncbi.nlm.nih.gov/snp.

[21] Wahbi K., Béhin A., Bécane H.M., Leturcq F., Cossée M., Laforêt P., Stojkovic T., Carlier P., Toussaint M., Gaxotte V. (2013). Dilated cardiomyopathy in patients with mutations in anoctamin 5. Int. J. Cardiol..

[22] Barresi R., Di Blasi C., Negri T., Brugnoni R., Vitali A., Felisari G., Salandi A., Daniel S., Cornelio F., Morandi L. (2000). Disruption of heart sarcoglycan complex and severe cardiomyopathy caused by beta sarcoglycan mutations. J. Med. Genet..

[23] Ben Hamida M., Ben Hamida C., Zouari M., Belal S., Hentati F. (1996). Limb-girdle muscular dystrophy 2C: Clinical aspects. Neuromuscul. Disord..

[24] Nigro V., Okazaki Y., Belsito A., Piluso G., Matsuda Y., Politano L., Nigro G., Ventura C., Abbondanza C., Molinari A.M. (1997). Identification of the Syrian hamster cardiomyopathy gene. Hum. Mol. Genet..

[25] Piccolo F., Roberds S.L., Jeanpierre M., Leturcq F., Azibi K., Beldjord C., Carrie A., Recan D., Chaouch M., Reghis A. (1995). Primary adhalinopathy: A common cause of autosomal recessive muscular dystrophy of variable severity. Nat. Genet..

[26] Tsubata S., Bowles K.R., Vatta M., Zintz C., Titus J., Muhonen L., Bowles N.E., Towbin J.A. (2000). Mutations in the human δ-sarcoglycan gene in familial and sporadic dilated cardiomyopathy. J. Clin. Investig..

[27] McNally E.M., Mestroni L. (2017). Dilated cardiomyopathy: Genetic determinants and mechanisms. Circ. Res..

[28] Sandra M., Maria Pia L., Stefano C., Pietro P., Crociani P., Aldo R., Giuseppe D.S., Massimo C. (2019). Emery-Dreifuss muscular dystrophy type 4: A new SYNE1 mutation associated with hypertrophic cardiomyopathy masked by a perinatal distress-related spastic diplegia. Clin. Case Rep..

[29] Zhuang Y., Gong Y.J., Zhong B.F., Zhou Y., Gong L. (2017). Bioinformatics method identifies potential biomarkers of dilated cardiomyopathy in a human induced pluripotent stem cell-derived cardiomyocyte model. Exp. Ther. Med..

[30] Murakami T., Hayashi Y.K., Noguchi S., Ogawa M., Nonaka I., Tanabe Y., Ogino M., Takada F., Eriguchi M., Kotooka N. (2006). Fukutin gene mutations cause dilated cardiomyopathy with minimal muscle weakness. Ann. Neurol..

[31] Anandan C., Cipriani M.A., Laughlin R.S., Niu Z., Milone M. (2018). Rhabdomyolysis and fluctuating asymptomatic hyperCKemia associated with CACNA1S variant. Eur. J. Neurol..

[32] Zuchner S., Mersiyanova I.V., Muglia M., Bissar-Tadmouri N., Rochelle J., Dadali E.L., Zappia M., Nelis E., Patitucci A., Senderek J. (2004). Mutations in the mitochondrial GTPase mitofusin 2 cause Charcot-Marie-Tooth neuropathy type 2A. Nat. Genet..

[33] Dorn G.W., Clark C.F., Eschenbacher W.H., Kang M.Y., Engelhard J.T., Warner S.J., Matkovich S.J., Jowdy C.C. (2011). MARF and Opa1 control mitochondrial and cardiac function in Drosophila. Circ. Res..

[34] Chen Y., Dorn G.W. (2013). PINK1-phosphorylated mitofusin 2 is a parkin receptor for culling damaged mitochondria. Science.

[35] Papanicolaou K.N., Khairallah R.J., Ngoh G.A., Chikando A., Luptak I., O’Shea K.M., Riley D.D., Lugus J.J., Colucci W.S., Lederer W.J. (2011). Mitofusin-2 Maintains Mitochondrial Structure and Contributes to Stress-Induced Permeability Transition in Cardiac Myocytes. Mol. Cell Biol..

[36] Stojkovic T., Richard P., Charron P., Rondeau S., JeanPierre M. (2014). Identification of both LMNA and SMCHD1 mutations in a case with overlapping phenotypes. Neuromuscul. Disord..

[37] Gal A., Inczedy-Farkas G., Pal E., Remenyi V., Bereznai B., Geller L., Szelid Z., Merkely B., Molnar M.J. (2015). The coexistence of dynamin 2 mutation and multiple mitochondrial DNA (mtDNA) deletions in the background of severe cardiomyopathy and centronuclear myopathy. Clin. Neuropathol..

[38] Watts G.D., Wymer J., Kovach M.J., Mehta S.G., Mumm S., Darvish D., Pestronk A., Whyte M.P., Kimonis V.E. (2004). Inclusion body myopathy associated with Paget disease of bone and frontotemporal dementia is caused by mutant valosin-containing protein. Nat. Genet..

[39] Mizuta K., Tsutsumi S., Inoue H., Sakamoto Y., Miyatake K., Miyawaki K., Noji S., Kamata N., Itakura M. (2007). Molecular characterization of GDD1/TMEM16E, the gene product responsible for autosomal dominant gnathodiaphyseal dysplasia. Biochem. Biophys. Res. Commun..

[40] Bolduc V., Marlow G., Boycott K.M., Saleki K., Inoue H., Kroon J., Itakura M., Robitaille Y., Parent L., Baas F. (2010). Recessive mutations in the putative calcium-activated chloride channel Anoctamin 5 cause proximal LGMD2L and distal MMD3 muscular dystrophies. Am. J. Hum. Genet..

[41] Hicks D., Sarkozy A., Muelas N., Koehler K., Huebner A., Hudson G., Chinnery P.F., Barresi R., Eagle M., Polvikoski T. (2011). A founder mutation in Anoctamin 5 is a major cause of limb-girdle muscular dystrophy. Brain.

[42] Campbell K.P., Kahl S.D. (1989). Association of dystrophin and an integral membrane glycoprotein. Nature.

[43] Yoshida M., Ozawa E. (1990). Glycoprotein complex anchoring dystrophin to sarcolemma. J. Biochem..

[44] Bonnemann C.G., Modi R., Noguchi S., Mizuno Y., Yoshida M., Gussoni E., McNally E.M., Duggan D.J., Angelini C., Hoffman E.P. (1995). Beta-sarcoglycan (A3b) mutations cause autosomal recessive muscular dystrophy with loss of the sarcoglycan complex. Nat. Genet..

[45] Lim L.E., Duclos F., Broux O., Bourg N., Sunada Y., Allamand V., Meyer J., Richard I., Moomaw C., Slaughter C. (1995). Beta-sarcoglycan: Characterization and role in limb-girdle muscular dystrophy linked to 4q12. Nat. Genet..

[46] Noguchi S., McNally E.M., Ben Othmane K., Hagiwara Y., Mizuno Y., Yoshida M., Yamamoto H., Bonnemann C.G., Gussoni E., Denton P.H. (1995). Mutations in the dystrophin-associated protein gamma-sarcoglycan in chromosome 13 muscular dystrophy. Science.

[47] Roberds S.L., Leturcq F., Allamand V., Piccolo F., Jeanpierre M., Anderson R.D., Lim L.E., Lee J.C., Tome F.M., Romero N.B. (1994). Missense mutations in the adhalin gene linked to autosomal recessive muscular dystrophy. Cell.

[48] Barresi R., Confalonieri V., Lanfossi M., Di Blasi C., Torchiana E., Mantegazza R., Jarre L., Nardocci N., Boffi P., Tezzon F. (1997). Concomitant deficiency of beta- and gamma-sarcoglycans in 20 alpha-sarcoglycan (adhalin)-deficient patients: Immunohistochemical analysis and clinical aspects. Acta Neuropathol..

[49] Ben Othmane K., Ben Hamida M., Pericak-Vance M.A., Ben Hamida C., Blel S., Carter S.C., Bowcock A.M., Petruhkin K., Gilliam T.C., Roses A.D. (1992). Linkage of Tunisian autosomal recessive Duchenne-like muscular dystrophy to the pericentromeric region of chromosome 13q. Nat. Genet..

[50] Van der Kooi A.J., de Visser M., van Meegen M., Ginjaar H.B., van Essen A.J., Jennekens F.G., Jongen P.J., Leschot N.J., Bolhuis P.A. (1998). A novel gamma-sarcoglycan mutation causing childhood onset, slowly progressive limb girdle muscular dystrophy. Neuromuscul. Disord..

[51] Townsend D., Yasuda S., McNally E., Metzger J.M. (2011). Distinct pathophysiological mechanisms of cardiomyopathy in hearts lacking dystrophin or the sarcoglycan complex. FASEB J..

[52] Bonnemann C.G., Wong J., Jones K.J., Lidov H.G., Feener C.A., Shapiro F., Darras B.T., Kunkel L.M., North K.N. (2002). Primary gamma-sarcoglycanopathy (LGMD 2C): Broadening of the mutational spectrum guided by the immunohistochemical profile. Neuromuscul. Disord..

[53] Lucioli S., Giusti B., Mercuri E., Vanegas O.C., Lucarini L., Pietroni V., Urtizberea A., Ben Yaou R., de Visser M., van der Kooi A.J. (2005). Detection of common and private mutations in the COL6A1 gene of patients with Bethlem myopathy. Neurology.

[54] Okada M., Kawahara G., Noguchi S., Sugie K., Murayama K., Nonaka I., Hayashi Y.K., Nishino I. (2007). Primary collagen VI deficiency is the second most common congenital muscular dystrophy in Japan. Neurology.

[55] Van der Kooi A.J., de Voogt W.G., Bertini E., Merlini L., Talim F.B., Ben Yaou R., Urtziberea A., de Visser M. (2006). Cardiac and pulmonary investigations in Bethlem myopathy. Arch. Neurol..

[56] Rutschow D., Bauer R., Gohringer C., Bekeredjian R., Schinkel S., Straub V., Koenen M., Weichenhan D., Katus H.A., Muller O.J. (2014). S151A delta-sarcoglycan mutation causes a mild phenotype of cardiomyopathy in mice. Eur. J. Hum. Genet..

[57] Zhou C., Li C., Zhou B., Sun H., Koullourou V., Holt I., Puckelwartz M.J., Warren D.T., Hayward R., Lin Z. (2017). Novel nesprin-1 mutations associated with dilated cardiomyopathy cause nuclear envelope disruption and defects in myogenesis. Hum. Mol. Genet..

[58] Lee J.H., Shin H.Y., Park H.J., Kim S.H., Kim S.M., Choi Y.C. (2017). Clinical, Pathologic, and Genetic Features of Collagen VI-Related Myopathy in Korea. J. Clin. Neurol..

[59] Tucker W.S., Hubbard W.H., Stryker T.D., Morgan S.W., Evans O.B., Freemon F.R., Theil G.B. (1982). A new familial disorder of combined lower motor neuron degeneration and skeletal disorganization. Trans. Assoc. Am. Physicians.

[60] Kimonis V.E., Kovach M.J., Waggoner B., Leal S., Salam A., Rimer L., Davis K., Khardori R., Gelber D. (2000). Clinical and molecular studies in a unique family with autosomal dominant limb-girdle muscular dystrophy and Paget disease of bone. Genet. Med..

[61] Kovach M.J., Waggoner B., Leal S.M., Gelber D., Khardori R., Levenstien M.A., Shanks C.A., Gregg G., Al-Lozi M.T., Miller T. (2001). Clinical delineation and localization to chromosome 9p13.3–p12 of a unique dominant disorder in four families: Hereditary inclusion body myopathy, Paget disease of bone, and frontotemporal dementia. Mol. Genet. Metab..

[62] Brody M.J., Vanhoutte D., Bakshi C.V., Liu R., Correll R.N., Sargent M.A., Molkentin J.D. (2019). Disruption of valosin-containing protein activity causes cardiomyopathy and reveals pleiotropic functions in cardiac homeostasis. J. Biol. Chem..

[63] Grossman T.R., Gamliel A., Wessells R.J., Taghli-Lamallem O., Jepsen K., Ocorr K., Korenberg J.R., Peterson K.L., Rosenfeld M.G., Bodmer R. (2011). Over-expression of DSCAM and COL6A2 cooperatively generates congenital heart defects. PLoS Genet..

[64] Connell P.S., Jeewa A., Kearney D.L., Tunuguntla H., Denfield S.W., Allen H.D., Landstrom A.P. (2019). A 14-year-old in heart failure with multiple cardiomyopathy variants illustrates a role for signal-to-noise analysis in gene test re-interpretation. Clin. Case Rep..

[65] Jones D.M., Lopes L., Quinlivan R., Elliott P.M., Khanji M.Y. (2019). Cardiac manifestations of McArdle disease. Eur. Heart J..

[66] Brodehl A., Gaertner-Rommel A., Milting H. (2018). Molecular insights into cardiomyopathies associated with desmin (DES) mutations. Biophys. Rev..

