# Supplementary material for: Genetic Modifiers of Hereditary Neuromuscular Disorders and Cardiomyopathy
Source: Cells. 2021 Feb 8;10(2):349. doi: 10.3390/cells10020349 (PMC7915259; doi:10.3390/cells10020349)
Supplement: Supplementary file 1 [file cells-10-00349-s001.pdf]

SUPPLEMENTAL MATERIAL

Genetic Modifiers of Hereditary Neuromuscular Disorders and Cardiomyopathies

Sholeh Bazrafshan[1], Hani Kushlaf [2], Mashhood Kakroo[1], John Quinlan[2], Richard C. Becker [1], and Sakthivel Sadayappan [1,*]

[1]Heart, Lung and Vascular Institute, Division of Cardiovascular Health and Disease, Department of Internal Medicine, University of Cincinnati College of Medicine, Cincinnati, OH 45267, USA; bazrafsh@ucmail.uc.edu (S.B.); kakroomd@ucmail.uc.edu (M.K.) and beckerrc@ucmail.uc.edu (R.C.B.)

[2]Department of Neurology and Rehabilitation, University of Cincinnati Gardner Neuroscience Institute, Neuromuscular Center; University of Cincinnati College of Medicine, Cincinnati, OH 45267, USA. kushlaha@ucmail.uc.edu (H.K.) and quinlajg@ucmail.uc.edu (J.Q.)

* Correspondence: sadayasl@ucmail.uc.edu (S.S.). Tel.: +1-513-558-7498

Subject # 1: Custom Limb-Girdle Muscular Dystrophy Panel - 35 genes (EGL Genetics, Dated 11/30/2017)

ANO5, CAPN3, CAV3, COL6A1, COL6A2, COL6A3, DAG1, DES, DMD, DNAJB6, DYSF, EMD, FHL1, FKRP, FKTN, GAA, GNE, ISPD, LMNA, MYOT, PLEC, POMGNT1, POMT1, POMT2, SGCA, SGCB, SGCD, SGCG, SMCHD1, SYNE1, TCAP, TNPO3, TRIM32, TTN and VCP

Subject # 2: Custom Limb-Girdle Muscular Dystrophy Panel - 35 genes (EGL Genetics, Dated 11/13/2015)

ANO5, CAPN3, CAV3, COL6A1, COL6A2, COL6A3, DAG1, DES, DMD, DNAJB6, DYSF, EMD, FHL1, FKRP, FKTN, GAA, GNE, ISPD, LMNA, MYOT, PLEC, POMGNT1, POMT1, POMT2, SGCA, SGCB, SGCD, SGCG, SMCHD1, SYNE1, SYNE2, TCAP, TNPO3, TRIM32 and TTN

Subject # 3: Custom Limb-Girdle Muscular Dystrophy Panel - 35 genes (EGL Genetics, Dated 9/3/2015)

ANO5, CAPN3, CAV3, COL6A1, COL6A2, COL6A3, DAG1, DES, DMD, DNAJB6, DYSF, EMD, FHL1, FKRP, FKTN, GAA, GNE, ISPD, LMNA, MYOT, PLEC, POMGNT1, POMT1, POMT2, SGCA, SGCB, SGCD, SGCG, SMCHD1, SYNE1, SYNE2, TCAP, TNPO3, TRIM32 and TTN

Subject # 4

1) Custom Limb-Girdle Muscular Dystrophy Panel – 34 genes (EGL Genetics, Dated 12/6/2016)

ANO5, CAPN3, CAV3, COL6A1, COL6A2, COL6A3, DAG1, DES, DMD, DNAJB6, DYSF, EMD, FHL1, FKRP, FKTN, GAA, GNE, ISPD, LMNA, MYOT, PLEC, POMGNT1, POMT1, POMT2, SGCA, SGCB, SGCD, SGCG, SMCHD1, SYNE1, TCAP, TNPO3, TRIM32 and TTN

2) Invitae Comprehensive Neuromuscular Disorders Panel - 122 genes (Inviate, Dated 4/4/2018)

ACTA1, AGRN, ALG14, ALG2, ANO5, ATP2A1, B3GALNT2, B4GAT1, BAG3, BIN1, CACNA1S, CAPN3, CAV3, CCDC78, CFL2, CHAT, CHKB, CHRNA1, CHRNB1, CHRND, CHRNE, CLCN1, CNTN1, COL12A1, COL6A1, COL6A2, COL6A3, COLQ, CPT2, CRYAB, DAG1, DES, DMD, DNAJB6, DNM2, DOK7, DPAGT1, DPM1, DPM2, DPM3, DYSF, EMD, FHL1, FKBP14, FKRP, FKTN, FLNC, GAA, GFPT1, GMPPB, GNE, HNRNPA2B1, HNRNPDL, ISPD, ITGA7, KBTBD13, KCNJ2, KLHL40, KLHL41, LAMA2, LAMB2, LAMP2, LARGE1, LDB3, LIMS2, LMNA, LMOD3, LRP4, MATR3, MEGF10, MTM1, MUSK, MYF6, MYH2, MYH7, MYL2, MYOT, MYPN, NEB, PLEC, PNPLA2, POMGNT1, POMGNT2, POMK, POMT1, POMT2, PREPL, RAPSN, RYR1, SCN4A, SELENON, SGCA, SGCB, SGCD, SGCG, SLC5A7, SMCHD1, SMN1, SMN2, SNAP25, SQSTM1, STAC3, STIM1, SUN1, SUN2, SYNE1, SYNE2, TAZ, TCAP, TIA1, TMEM43, TMEM5, TNNT1, TNPO3, TOR1AIP1, TPM2, TPM3, TRAPPC11, TRIM32, TTN, VCP and VMA21

Subject # 5 Invitae Comprehensive Neuropathies Panel - 72 genes (Inviate, Dated 9/28/2018)

AARS, AIFM1, ATL1, ATL3, ATP7A, BICD2, BSCL2, CHCHD10, DCTN1, DNAJB2, DNM2, DNMT1, DST, DYNC1H1, EGR2, FAM134B, FBXO38, FGD4, FIG4, GAN, GARS, GDAP1, GJB1, GNB4, HARS, HINT1, HSPB1, HSPB8, IGHMBP2, IKBKAP, INF2, KIF1A, LITAF, LMNA, LRSAM1, MED25, MFN2, MORC2, MPZ, MTMR2, NDRG1, NEFL, NGF, NTRK1, PDK3, PLEKHG5, PMP22, PRPS1, PRX, RAB7A, REEP1, SBF2, SCN11A, SCN9A, SH3TC2, SIGMAR1, SLC25A46, SLC52A2, SLC52A3, SLC5A7, SPG11, SPTLC1, SPTLC2, TFG, TRIM2, TRPV4, TTR, UBA1, VAPB, VRK1, WNK1 and YARS

Subject # 6: Custom Limb-Girdle Muscular Dystrophy Panel - 35 genes (EGL Genetics, Dated 1/7/2019)

ANO5, CAPN3, CAV3, COL6A1, COL6A2, COL6A3, DAG1, DES, DMD, DNAJB6, DYSF, EMD, FHL1, FKRP, FKTN, GAA, GNE, ISPD, LMNA, MYOT, PLEC, POMGNT1, POMT1, POMT2, SGCA, SGCB, SGCD, SGCG, SMCHD1, SYNE1, TCAP, TNPO3, TRIM32, TTN and VCP

Subject # 7: Invitae Comprehensive Neuropathies Panel - 72 genes (Inviate, Dated 11/16/2018)

AARS, AIFM1, ATL1, ATL3, ATP7A, BICD2, BSCL2, CHCHD10, DCTN1, DNAJB2, DNM2, DNMT1, DST, DYNC1H1, EGR2, ELP1, FBXO38, FGD4, FIG4, GAN, GARS, GDAP1, GJB1, GNB4, HARS, HINT1, HSPB1, HSPB8, IGHMBP2, INF2, KIF1A, LITAF, LMNA, LRSAM1, MED25, MFN2, MORC2, MPZ, MTMR2, NDRG1, NEFL, NGF, NTRK1, PDK3, PLEKHG5, PMP22, PRPS1, PRX, RAB7A, REEP1, RETREG1, SBF2, SCN11A, SCN9A, SH3TC2, SIGMAR1, SLC25A46, SLC52A2, SLC52A3, SLC5A7, SPG11, SPTLC1, SPTLC2, TFG, TRIM2, TRPV4, TTR, UBA1, VAPB, VRK1, WNK1 and YARS

Subject # 8: Custom Limb-Girdle Muscular Dystrophy Panel - 35 genes (EGL Genetics, Dated 3/30/3018)

ANO5, CAPN3, CAV3, COL6A1, COL6A2, COL6A3, DAG1, DES, DMD, DNAJB6, DYSF, EMD, FHL1, FKRP, FKTN, GAA, GNE, ISPD, LMNA, MYOT, PLEC, POMGNT1, POMT1, POMT2, SGCA, SGCB, SGCD, SGCG, SMCHD1, SYNE1, TCAP, TNPO3, TRIM32, TTN and VCP

Subject # 9: Expanded Neuromuscular Disorders: Deletion/Duplication Panel with comparative genomic hybridization array - 47 genes (EGL Genetics, Dated 11/21/2014)

ACTA1, AMPD1, AMPD3, ANO5, CAPN3, CAV3, COL6A1, COL6A2, COL6A3, DES, DMD, DYSF, EMD, FKRP, FKTN, GAA, GNE, ISPD, ITGA7, LAMA2, LARGE, LDB3, LMNA, MYOT, NEB, PLEC, PMM2, POMGNT1, POMT1, POMT2, PYGM, RYR1, RYR2, SEPN1, SGCA, SGCB, SGCD, SGCE, SGCG, SIL1, TCAP, TNNI2, TNNT1, TPM2, TPM3, TRIM32 and TTN

Subject # 10

1. **Custom Limb-Girdle Muscular Dystrophy Panel - 35 genes (EGL Genetics, Dated 10/5/2015)**

ANO5, CAPN3, CAV3, COL6A1, COL6A2, COL6A3, DAG1, DES, DMD, DNAJB6, DYSF, EMD, FHL1, FKRP, FKTN, GAA, GNE, ISPD, LMNA, MYOT, PLEC, POMGNT1, POMT1, POMT2, SGCA, SGCB, SGCD, SGCG, SMCHD1, SYNE1, SYNE2, TCAP, TNPO3, TRIM32 and TTN

2. **Comprehensive Neuromuscular Disorder Panel - 100 genes (PerkinElmer, Dated 8/28/2019)**

ACTA1, ADSSL1, AMPD1, ANO5, ATP2A1, B3GALNT2, BAG3, BIN1, BVES, CAPN3, CAV3, CFL2, CHAT, CHRNA1, CHRNB1, CHRND, CHRNE, CLCN1, CNBP, COL12A1, COL6A1, COL6A2, COL6A3, COLQ, CRYAB, DAG1, DES, DMD, DMPK, DNAJB6, DNM2, DOK7, DPM3, DYSF, EMD, FHL1, FKRP, FKTN, FLNC, GAA, GMPPB, GNE, GOSR2, HNRNPA1, HNRNPDL, HSPG2, IGHMBP2, ISPD, ITGA7, LAMA2, LARGE, LDB3, LIMS2, LMNA, LYST, MATR3, MRC11, MTM1, MTMR14, MUSK, MYH2, MYH7, MYOT, NEB, PABPN1, PLEC1, PNPLA2, POGLUT1, POMGNT1, POMK, POMT1, POMT2, PTRF, PYGM, RAPSN, RNPA2, RYR1, SCN4A, SELENON, SEPN1, SGCA, SGCB, SGCD, SGCE, SGCG, SIL1, SMCHD1, SYNE1, SYNE2, TCAP, TIA1, TNNT1, TNPO3, TOR1AIP1, TPM2, TPM3, TRAPPC11, TRIM32, TTN and VCP

Subject # 11: Custom Limb-Girdle Muscular Dystrophy Panel - 34 genes (EGL Genetics, Dated 6/20/2016)

ANO5, CAPN3, CAV3, COL6A1, COL6A2, COL6A3, DAG1, DES, DMD, DNAJB6, DYSF, EMD, FHL1, FKRP, FKTN, GAA, GNE, ISPD, LMNA, MYOT, PLEC, POMGNT1, POMT1, POMT2, SGCA, SGCB, SGCD, SGCG, SMCHD1, SYNE1, TCAP, TNPO3, TRIM32 and TTN

Subject # 12: Custom Limb-Girdle Muscular Dystrophy Panel - 34 genes (EGL Genetics, Dated 12/28/2016)

ANO5, CAPN3, CAV3, COL6A1, COL6A2, COL6A3, DAG1, DES, DMD, DNAJB6, DYSF, EMD, FHL1, FKRP, FKTN, GAA, GNE, ISPD, LMNA, MYOT, PLEC, POMGNT1, POMT1, POMT2, SGCA, SGCB, SGCD, SGCG, SMCHD1, SYNE1, TCAP, TNPO3, TRIM32 and TTN

Subject # 13: Custom Limb-Girdle Muscular Dystrophy Panel - 35 genes (EGL Genetics, Dated 7/23/2015)

ANO5, CAPN3, CAV3, COL6A1, COL6A2, COL6A3, DAG1, DES, DMD, DNAJB6, DYSF, EMD, FHL1, FKRP, FKTN, GAA, GNE, ISPD, LMNA, MYOT, PLEC, POMGNT1, POMT1, POMT2, SGCA, SGCB, SGCD, SGCG, SMCHD1, SYNE1, SYNE2, TCAP, TNPO3, TRIM32 and TTN

Subject # 14: Custom Limb-Girdle Muscular Dystrophy Panel - 34 genes (EGL Genetics, Dated 1/5/2017)

ANO5, CAPN3, CAV3, COL6A1, COL6A2, COL6A3, DAG1, DES, DMD, DNAJB6, DYSF, EMD, FHL1, FKRP, FKTN, GAA, GNE, ISPD, LMNA, MYOT, PLEC, POMGNT1, POMT1, POMT2, SGCA, SGCB, SGCD, SGCG, SMCHD1, SYNE1, TCAP, TNPO3, TRIM32 and TTN
